# The Association of Pregnancy-induced Hypertension with Bronchopulmonary Dysplasia – A Retrospective Study Based on the Korean Neonatal Network database

**DOI:** 10.1038/s41598-020-62595-7

**Published:** 2020-03-27

**Authors:** Seung Hyun Shin, Seung Han Shin, Seh Hyun Kim, Yoo-Jin Kim, Hannah Cho, Ee-Kyung Kim, Han-Suk Kim

**Affiliations:** Department of Pediatrics, Seoul National University College of Medicine, Seoul, South Korea

**Keywords:** Pre-eclampsia, Respiratory tract diseases, Paediatric research

## Abstract

The prevalence of pregnancy-induced hypertension (PIH) and preeclampsia (PE) are 5–10% and 2–4%, respectively. PIH might affect angiogenesis in preterm neonates, but its association with bronchopulmonary dysplasia (BPD) remains controversial. This study evaluated the association between PIH and BPD in very low-birth weight infants. We retrospectively analysed the maternal, perinatal, and neonatal data of preterm infants born before 30 weeks of gestation, selected from the nationwide registry of very low-birth weight infants, between January 2013 and December 2014. As a result, 1,624 infants without maternal PIH (gestational age: 27.3 ± 1.8 weeks) and 203 infants with maternal PIH (28.0 ± 1.4 weeks, p < 0.001) were included. Birth weight was higher in the non-PIH group, compared with the PIH group (1027.4 ± 250.2 vs. 876.4 ± 261.5 g, p < 0.001). Multivariate logistic regression showed that PIH was associated with BPD (adjusted OR 1.474, 95% confidence interval 1.025–2.121), after adjusting for confounders, including small-for-gestation age (SGA). The result of present study is consistent with the current concept of BPD as an early form of pulmonary vascular disease, for both PIH and BPD are attributed by abnormal vascular formation.

## Introduction

Bronchopulmonary dysplasia (BPD) is the most common complication of preterm birth among very low-birth weight (VLBW) infants. Although the progression of prematurity care, including the use of antenatal steroids and surfactant therapy, has improved neonatal outcomes, the incidence of BPD has not decreased with the increased survival of preterm infants^1–4^. These extremely preterm infants present with arrest in the development of the alveolar-capillary unit, characterized by enlarged and simplified alveolar structure, prominent interstitial fibrosis, and abnormal pulmonary vasculature^5^. For angiogenesis and alveolar development are interactive in the foetal lung, BPD has been recently recognized as a manifestation of vascular disease of the lungs in this population^6^.

The prevalence of pregnancy-induced hypertension (PIH) and preeclampsia (PE) are 5–10% and 2–4%, respectively^7–10^. Although the pathophysiology of PIH and PE are not established, inadequate placental implantation and abnormal vascularization might play certain roles in the development of PIH^11^. Several maternal angiogenic factors and anti-angiogenic factors regulate placental growth and vascular development in PE^12–14^. Inappropriate vascular endothelial growth factor (VEGF) signalling could also alter vascular development and reactivity in foetus, leading to neonatal pulmonary vascular disease^15^. In a clinical study, the pulmonary outcomes of preterm infants were predicted by measurements of the placental growth factor from the mother^16^. Since both PIH and BPD are associated with abnormal angiogenesis, there has been efforts to evaluate the association between PIH and BPD in preterm infants. A small prospective study showed that moderate to severe BPD was more prevalent in preterm infants born at less than 32 weeks of gestation, to mothers with PE^17^. A recent meta-analysis and international cohort study also reported that PIH was associated with BPD^18,19^.

On the other hand, a meta-analysis of three cohorts in Austria reported that PE was not associated with BPD^20^. Another large population-based study also reported that preeclampsia decreased the risk for BPD in VLBW infants^21^. This study demonstrated a negative association between PE and BPD only in the group with a gestational age (GA) of more than 31 weeks. However, as hypertensive disorders in pregnancy are greatly associated with small-for-gestational age (SGA) infants^22^, studies using birth weight-based registries should be interpreted with caution, because more mature but smaller infants could be included in the registry.

This retrospective cohort study selected and analysed infants born at less than 30 weeks of gestation, from a nationwide registry database of VLBW infants in Korea, to investigate the association between PIH and BPD.

## Methods

The Korean Neonatal Network (KNN) is a national prospective registry of VLBW infants (birth weight < 1,500 g) born in the Republic of Korea, covering more than 70% of the overall births of VLBW infants in Korea^23^. The KNN registry was approved by the institutional review board (IRB) at each participating hospital, and informed consent was obtained from the parents of all infants at enrollment by the NICUs participating in the KNN. All methods were carried out in accordance with the IRB-approved protocol and in compliance with relevant guidelines and regulations. The definitions of the data were guided by the manual of operation of the KNN and the data registered in the KNN database comprises the antenatal and perinatal histories, postnatal morbidities, and clinical outcomes evaluated during the hospital stay using a standardized electronic case-report form. Altogether 3,507 VLBW infants were born and registered with the KNN database from January 2013 to December 2014, and 2,276 infants who were born at less than 30 weeks of gestation were enrolled in the study. Infants with congenital anomalies, those born to mothers with chronic hypertension, infants without BPD data, and infants who died before 36 weeks of post-menstrual age (PMA) were excluded from the study.

PIH was defined as newly diagnosed hypertension in a pregnant woman after 20 weeks of gestation, where systolic blood pressure was ≥140 mmHg and/or diastolic blood pressure was ≥90 mmHg. BPD was defined as the need for supplemental oxygen or positive pressure support at 36 weeks of PMA. This corresponds with moderate or severe BPD, according to the severity-based definition for BPD provided by the National Institute of Health consensus^24^. Respiratory distress syndrome (RDS) was defined by clinical diagnosis and required surfactant therapy. Intraventricular haemorrhage (IVH) was defined by the Papile criteria, using cranial ultrasonography^25^. Necrotizing enterocolitis (NEC) was defined according to Bell’s criteria (stage 2 or higher)^26^. Retinopathy of prematurity (ROP) was defined according to the international classification of ROP^27^. SGA was defined as birth weight lower than the third percentile for GA, according to Fenton’s growth charts^28^. The study was approved by the Institutional Review Board of Seoul National University Hospital (1701-024-821).

The Chi-squared test was used for comparing categorical variables and the Student’s two-tailed *t*-test was used for analysing the continuous variables between groups. Univariate and multivariate logistic regression analyses were used to investigate the association between PIH and BPD, after adjusting for potential confounders. Adjusted odds ratios, with a 95% confidence interval (CI) were obtained, to assess the magnitude of the association between various factors and BPD. Subgroup analysis according to the GA (<27 weeks and 27-29 weeks) was done to figure out whether the effects of PIH on BPD vary between different age groups. Sensitivity analysis was conducted with an extended population including death before PMA 36 weeks using a composite outcome of BPD and death to investigate the association with PIH. Data are presented as the mean ± standard deviation. Statistical analyses were performed with STATA 11.0 (Stata Corp, College Station, Tex., USA).

## Results

**Figure 1:**
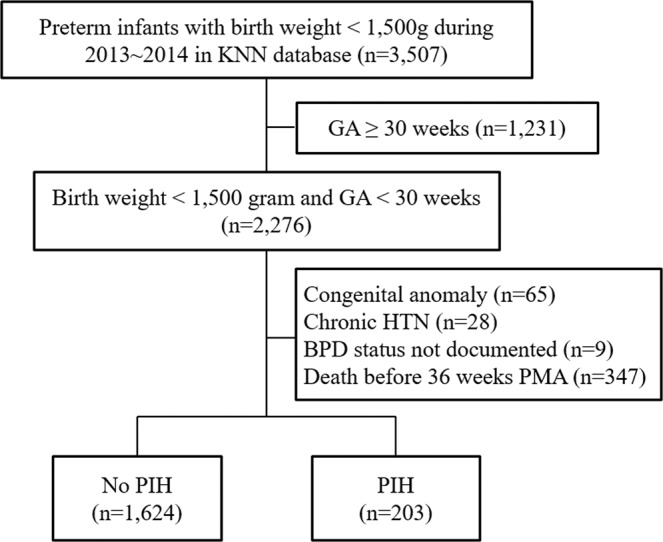
Flow chart of the study population. Among 3,507 infants registered, 2,276 infants who were born at less than 30 weeks of gestation were enrolled and 1,827 were analysed. BPD: bronchopulmonary dysplasia, GA: gestational age, HTN: hypertension, KNN: Korean Neonatal Network, PIH: pregnancy-induced hypertension, PMA: postmenstrual age.

Of the 3,507 infants registered with the KNN database during the study period, 1,231 infants were born at or more than 30 weeks of gestation, 28 infants were born to mothers with chronic hypertension and 65 infants had a congenital anomaly (Fig. 1). Nine infants without BPD data and 347 infants who died before 36 weeks of PMA were also excluded. Of the remaining 1,827 infants, 203 (11.1%) infants were born to mothers with PIH and 1,624 (88.9%) infants were born to mothers without hypertension.

The GA of the PIH group was higher than that of the non-PIH group (27.3 ± 1.8 versus 28.0 ± 1.4 weeks, p < 0.001) (Table 1). Birth weight was lower in the PIH group than that in the non-PIH group (876.4 ± 261.5 versus 1027.4 ± 250.2 g, p < 0.001). Infants who were SGA were more common in the PIH group than the non-PIH group (1.4% vs. 9.9%, p < 0.001). Infants in the PIH group received more prenatal steroid therapy and were mostly delivered through a Cesarean section. On the other hand, multiple births, histologic chorioamnionitis (hCAM), and preterm premature rupture of the membrane were more prevalent in the non-PIH group. Severe IVH (≥grade 3) was significantly lower in the PIH group (10.6% versus 5.4%, p = 0.018) and the duration of total parenteral nutrition (TPN) was significantly longer in the PIH group (33.9 ± 28.6 versus 38.5 ± 28 days, p = 0.031) (Table 2). There were no differences in the duration of invasive ventilation or hospital stay between the non-PIH and PIH groups. The incidence of BPD (41.4% in the non-PIH group versus 44.8% in the PIH group) was not significantly different between the two groups.

**Table 1:** Demographics of study population.

	non-PIH (n = 1,624)	PIH (n = 203)	p value
GA (weeks)	27.3 ± 1.8	28.0 ± 1.4	<0.001
Birthweight (grams)	1027.4 ± 250.2	876.4261.5	<0.001
Prenatal steroid	1294 (81)	176 (87.6)	0.026
SGA	23 (1.4)	20 (9.9)	<0.001
C/S	1084 (66.8)	191 (94.1)	<0.001
Male	850 (52.3)	94 (46.3)	0.118
AS 1 min	4.3 ± 1.9	4.0 ± 1.8	0.011
AS 5 min	6.5 ± 1.7	6.5 ± 1.7	0.948
Oligohydramnios	197 (13.4)	21 (10.9)	0.366
hCAM	614 (44.9)	22 (12.3)	<0.001
Multiple birth	551 (33.9)	26 (12.8)	<0.001
PPROM	785 (48.7)	15 (7.4)	<0.001

Values are expressed as N (%) or Mean ± SD; PIH, pregnancy induced hypertension; GA, gestational age; SGA, small for gestational age; C/S, Cesarean section; AS, Apgar score; hCAM, histologic chorioamnionitis; PPROM, preterm premature rupture of membrane.

**Table 2:** Neonatal morbidities of study population.

	non-PIH (n = 1,624)	PIH (n = 203)	p value
RDS	1522 (93.7)	191 (94.1)	1.000
Treated PDA	828 (51)	107 (52.7)	0.656
IVH ≥ grade 3	172 (10.6)	11 (5.4)	0.018
PVL	182 (11.2)	16 (7.9)	0.187
NEC	100 (6.2)	19 (9.4)	0.095
Treated ROP	254 (22)	34 (25.2)	0.385
BPD	673 (41.4)	91 (44.8)	0.366
Postnatal steroid	608 (37.4)	70 (34.5)	0.441
Sepsis	427 (26.3)	58 (28.6)	0.500
Ventilator duration (days)	24.1 ± 31.9	25.6 ± 36	0.547
TPN duration (days)	33.9 ± 28.6	38.5 ± 28	0.031
Hospital days	87 ± 38.3	90.8 ± 42.4	0.185

Values are expressed as N (%) or Mean ± SD; PIH, pregnancy induced hypertension; RDS, respiratory distress syndrome; PDA, patent ductus arteriosus; IVH, intraventricular hemorrhage; PVL, periventricular leukomalacia; NEC, necrotizing enterocolitis; ROP, retinopathy of prematurity; BPD, bronchopulmonary dysplasia; TPN, total parental nutrition.

Univariate and multivariate logistic regression analyses were performed to evaluate the risk factors for BPD. In the univariate analysis, lower GA at birth, SGA, RDS, male sex, hCAM, and treated patent ductus arteriosus (PDA) were associated with BPD (Table 3). Although PIH was not associated with BPD on the univariate analysis, PIH was associated with BPD (adjusted OR 1.474, 95% CI 1.025–2.121) along with GA, SGA, male sex, hCAM and treated PDA on the multivariate analysis. The subgroup analyses according to GA stratification were performed and multivariate analysis showed that there was an association between PIH and BPD in VLBW infants with 27–29 weeks of gestation (adjusted OR 1.626, 95% CI 1.075–2.460), but not in the other GA groups (Table 4).

**Table 3:** Univariate and multivariate conditional logistic regression analysis of BPD.

	OR	95% CI	p-value	aOR^¶^	95% CI	p-value
GA (week)	0.646	[0.608, 0.686]	<0.001	0.673	[0.627, 0.722]	<0.001
SGA	5.454	[2.600, 11.441]	<0.001	5.647	[2.417, 13.194]	<0.001
RDS	3.392	[2.093, 5.499]	<0.001	1.617	[0.961, 2.719]	0.070
Male	1.256	[1.042, 1.513]	0.017	1.393	[1.111, 1.747]	0.004
hCAM	1.597	[1.301, 1.962]	<0.001	1.528	[1.206, 1.936]	<0.001
Treated PDA	2.788	[2.298, 3.382]	<0.001	2.299	[1.823, 2.898]	<0.001
PIH	1.148	[0.856, 1.540]	0.357	1.474	[1.025, 2.121]	0.036

BPD, bronchopulmonary dysplasia; GA, gestational age; SGA, small for gestational age; RDS, respiratory distress syndrome; hCAM, histologic chorioamnionitis; PDA, patent ductus arteriosus; PIH, pregnancy induced hypertension; ^¶^adjusted for GA, SGA, RDS, sex, hCAM, treated PDA and PIH.

**Table 4:** Subgroup analysis of association of pregnancy induced hypertension and BPD.

	non-PIH	PIH	p-value	aOR^¶^	95% CI	p-value
GA < 27 weeks	385/637 (60.4)	27/43 (62.8)	0.872	1.054	[0.495, 2.243]	0.892
GA 27–29 weeks	288/987 (29.2)	64/160 (40)	0.007	1.626	[1.075, 2.460]	0.021

BPD, bronchopulmonary dysplasia; PIH, pregnancy induced hypertension; GA, gestational age; ^¶^adjusted for gestational age, respiratory distress syndrome, sex, histologic chorioamnionitis, treated patent ductus arteriosus and PIH.

Multivariate analyses with a population including infants who died before 36 weeks of PMA were further studied as a sensitivity analysis to evaluate the association of PIH with the composite outcome of BPD or death at 36 weeks of PMA (Table S2). PIH was associated with BPD or death at 36 weeks of PMA (adjusted OR 1.428, 95% CI 1.015–2.008).

## Discussion

In this study, PIH was associated with BPD in the VLBW infants, who were born at less than 30 weeks of gestation, after adjusting for other risk factors. Impaired pulmonary vascular growth by altered signalling of angiogenic or antiangiogenic factors derived from mothers with hypertension may play a role in the pathogenesis of BPD^29^. Although how maternal angiogenic and anti-angiogenic factors influence the development of foetal lungs is not fully understood, elevation in anti-angiogenic factors, such as soluble fms-like tyrosine kinase-1 and soluble endoglin in the placenta and cord blood of a mother with PE might affect angiogenesis in foetal lungs with low levels of VEGF and placental growth factor^30,31^. Earlier studies from our group also demonstrated that increased soluble endoglin (an antiangiogenic factor) in cord blood was associated with the development of BPD in preterm infants born to mothers with PE^32^.

However, the results of several studies on the association of PIH or PE with BPD are conflicting. A population-based study analysing 5,753 VLBW infants by Yen *et al*. reported that foetal exposure to maternal PE was associated with a reduced risk of BPD^21^. A meta-analysis of 1,268 infants from three Victorian Infant Collaborative Study cohorts showed that PE did not influence the risk of BPD in extremely low-birth weight infants^20^. Results from these studies should be interpreted with caution due to several reasons. In the study by Yen *et al*., the study population included VLBW infants and was not defined according to the GA. Therefore, relatively smaller, mature babies were mostly included in the study population, especially in the PE group with a higher prevalence of SGA (73.3%). The protective effect of PE on BPD was shown only in the subgroup with a GA of 31~34 weeks, while the number of mature babies with SGA as a confounder would be higher in the VLBW population.

Studies with a GA-based population have yielded results that are different from earlier studies. An international cohort study comprising international neonatal (iNeo) databases based on GA analysed 28,092 preterm neonates born at 24 to 28 weeks of gestation and reported that the risk for BPD increased in infants born to mothers with hypertensive disorders of pregnancy^18^. A meta-analysis by Razak *et al*. reported that PIH was associated with BPD in the subpopulation of neonates born at <29 weeks of gestation^19^.

PIH is associated with babies that are small for the GA at birth^33,34^. The baby’s size and GA both have a significantly inverse relationship with BPD^35^. These associations were demonstrated in the present study as well. Therefore, the “size for gestational age” should be adjusted or GA-based population determination should be considered, while analysing the association between PIH and BPD, similar to the iNeo study or the study by Razak *et al*.^18,19^. To elucidate the association between BPD and PIH amidst these confounders, infants with a GA of 30 weeks or more were excluded from the study population, where SGA babies would be preferentially included in the weight-based registration. Sensitivity analysis among the population containing those who died before 36 weeks of PMA showed that composite outcome of BPD or death was also associated with PIH. Among the 347 infants who died before 36 weeks of PMA, cardiorespiratory failure was a leading cause of death, but there was no significant difference in the proportion of cardiorespiratory causes of death between the PIH and non-PIH groups (Table S3).

In the subgroup analysis, more mature infants with a GA 27~29 weeks were influenced by PIH in the development of BPD. A higher incidence of BPD in the subgroup with a GA < 27 weeks compared to those with a GA 27~29 weeks (60.4~62.8% vs. 29.2~40%, respectively) might reflect the vulnerability of the less mature lungs, and the alveolar-capillary structure might be arrested in the earlier phase of development in this population^5^. Moreover, immature infants experience various kinds of stress during the neonatal period, such as oxygen stress, inflammation, and hypoxia. Since BPD is an entity with a multifactorial pathogenesis^5^, there could be many factors that contribute to the development of BPD other than imbalanced vascular growth influenced by the maternal side. Differences in the association of maternal hypertensive disorder among GA subgroups were also well demonstrated in the large cohort study from Israel Neonatal Network^36^.

In conclusion, PIH was associated with BPD in VLBW infants, who were born before 30 weeks of gestation. The utilization of a nationwide registration database of VLBW infants in Korea was a strength of this study. This study’s results are consistent with the current concept that BPD is an early manifestation of pulmonary vascular disease.

## Supplementary information

Supplementary tables.

## Data Availability

According to the Korean Neonatal Network (KNN) Publication Ethics Policy, all registered data is confidential and is only available to researchers who have permission of access for the research activities. The dataset generated and analysed are not publicly available, but are available from the corresponding author on reasonable request.
