# Supplementary material for: The Association of Pregnancy-induced Hypertension with Bronchopulmonary Dysplasia – A Retrospective Study Based on the Korean Neonatal Network database
Source: Sci Rep. 2020 Mar 27;10:5600. doi: 10.1038/s41598-020-62595-7 (PMC7101434; doi:10.1038/s41598-020-62595-7)
Supplement: Supplementary file 1 — Supplementary tables. [file 41598_2020_62595_MOESM1_ESM.pdf]

The Association of Pregnancy-induced Hypertension with Bronchopulmonary Dysplasia – A Retrospective Study Based on the Korean Neonatal Network database

Authors: Seung Hyun Shin, M.D., Seung Han Shin, M.D.*, Seh Hyun Kim, M.D., Yoo-Jin Kim, Hannah Cho M.D., Ee-Kyung Kim, M.D., Han-Suk Kim, M.D.

Table S1. Characteristics of population including death before 36 weeks of postmenstrual age

	non-PIH (n=1,939)	PIH (n=235)	p-value
GA (week)	27±1.9	27.7±1.6	<0.001
Birthweight (gram)	985.1±266.4	847.4±264.7	<0.001
Prenatal steroid	1,500 (78.9)	201 (86.3)	0.007
SGA	38 (2.0)	27 (11.5)	<0.001
C/S	1288 (66.4)	216 (91.9)	<0.001
Male	1017 (52.5)	108 (46)	0.062
Oligohydramnios	236 (13.7)	27 (12.1)	0.603
hCAM	720 (44.5)	25 (12.1)	<0.001
Multiple birth	687 (35.4)	31 (13.2)	<0.001
PPROM	916 (47.6)	17 (7.3)	<0.001
RDS	1827 (94.2)	223 (94.9)	0.767
Treated PDA	951 (49.1)	125 (53.2)	0.241
BPD	674 (41.5)	91 (44.8)	0.366
Postnatal steroid	653 (33.7)	75 (31.9)	0.609
Death	315 (16.3)	32 (13.6)	0.346
BPD or death	988 (51)	123 (52.3)	0.730
Ventilator duration (days)	22.4±30	24.5±36.8	0.338

Values are expressed as N (%) or Mean±SD; PIH, pregnancy induced hypertension; GA, gestational age; SGA, small for gestational age; C/S, cesarean section; hCAM, histologic chorioamnionitis; PPROM, Preterm Premature Rupture of Membranes; RDS, respiratory distress syndrome; PDA, patent ductus arteriosus; BPD, bronchopulmonary dysplasia

Table S2. Univariate and multivariate logistic regression analysis of BPD or death in the population including death before 36 weeks of postmenstrual age

	OR	95% CI					p-value	aOR[¶]	95% CI					p-value
GA (week)	0.588	[	0.556	,	0.622	]	<0.001	0.593	[	0.556	,	0.632	]	<0.001
SGA	6.216	[	3.059	,	12.632	]	<0.001	6.415	[	2.846	,	14.458	]	<0.001
RDS	3.340	[	2.204	,	5.061	]	<0.001	1.618	[	1.003	,	2.609	]	0.049
Male	1.212	[	1.024	,	1.435	]	0.025	1.326	[	1.073	,	1.637	]	0.009
hCAM	1.450	[	1.202	,	1.750	]	<0.001	1.243	[	0.996	,	1.551	]	0.054
Treated PDA	1.999	[	1.685	,	2.371	]	<0.001	1.665	[	1.346	,	2.059	]	<0.001
PIH	1.057	[	0.806	,	1.386	]	0.688	1.428	[	1.015	,	2.008	]	0.041

BPD, bronchopulmonary dysplasia; OR, odds ratio; GA, gestational age; SGA, small for gestational age; RDS, respiratory distress syndrome; hCAM, histologic chorioamnionitis; PDA, patent ductus arteriosus; PIH, pregnancy induced hypertension; ¶ adjusted for GA, SGA, RDS, sex, hCAM, treated PDA and PIH

Table S3. Cause of death before 36 weeks of postmenstrual age

	non-PIH (n=315)	PIH (n=32)
Cardiorespiratory	140 (45.2)	11 (34.4)
Neurological	42 (13.6)	2 (6.3)
Infection	54 (17.4)	9 (28.1)
Gastrointestinal	42 (13.6)	6 (18.8)
Others	37 (11.8)	4 (12.5)

PIH, pregnancy induced hypertension; There was no statistical difference between two groups
